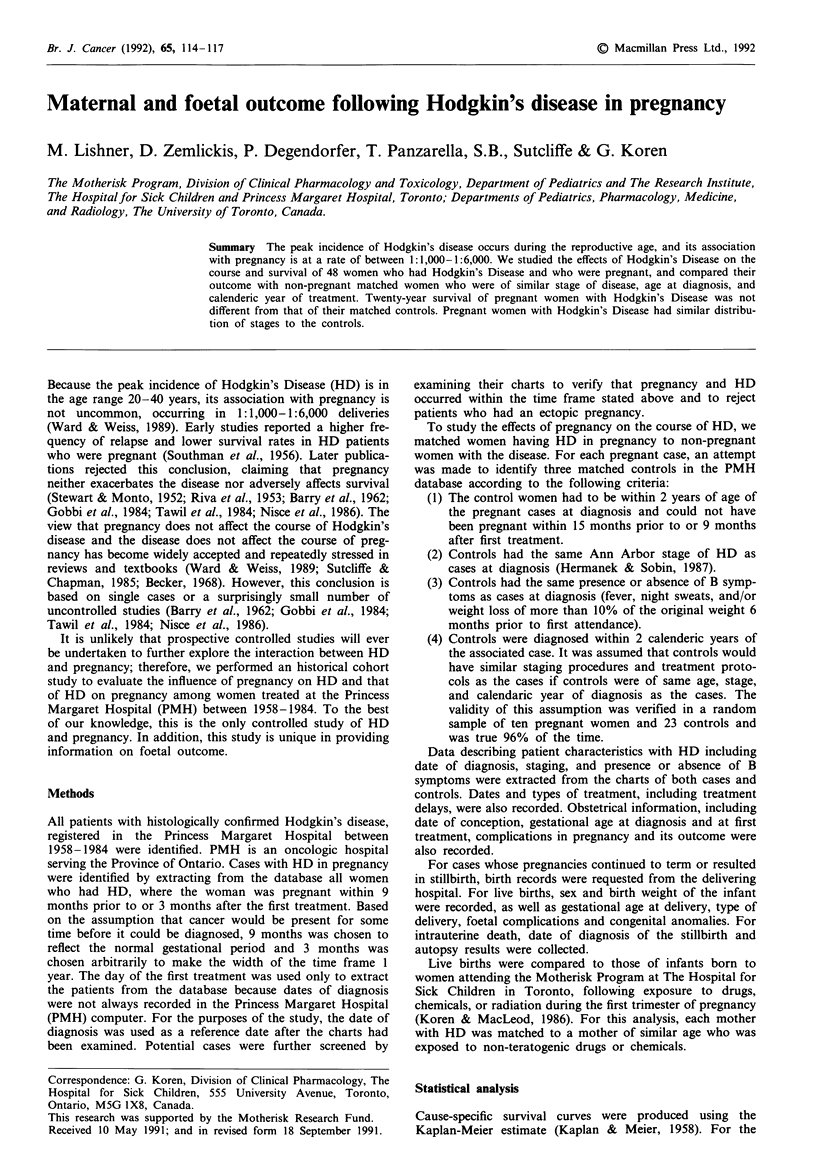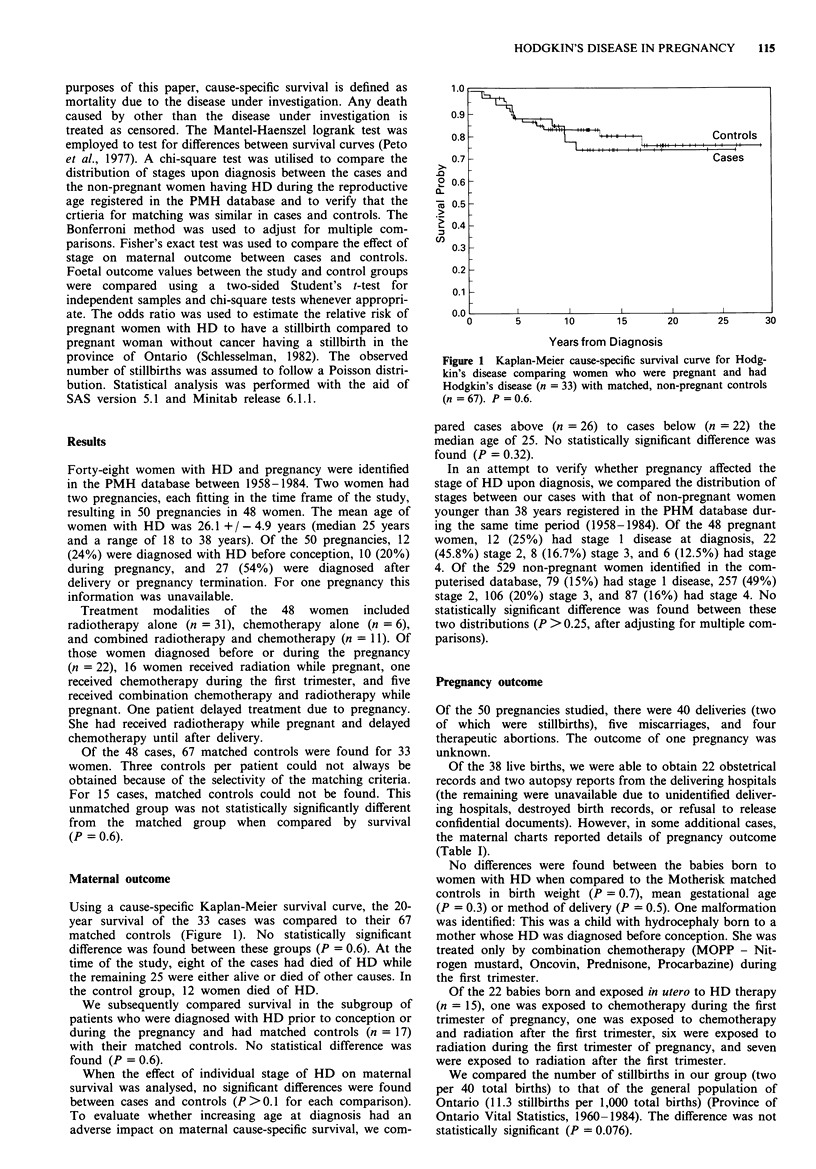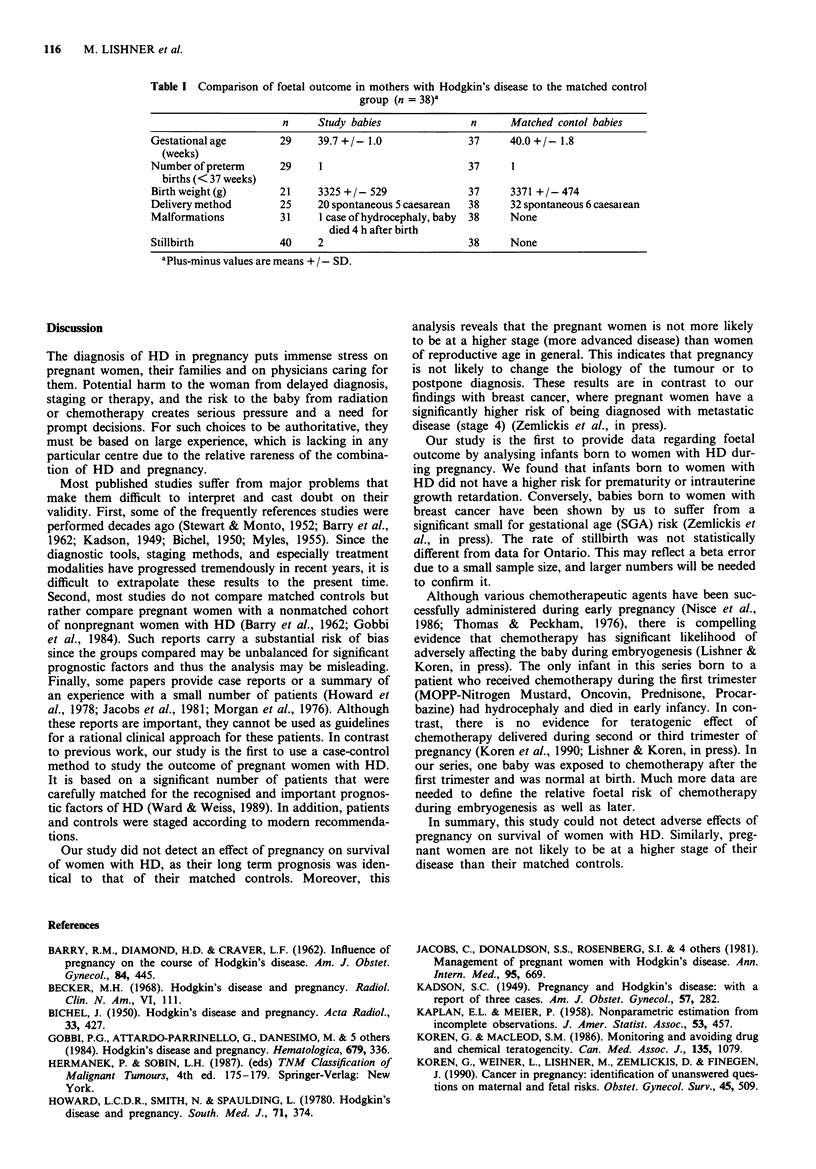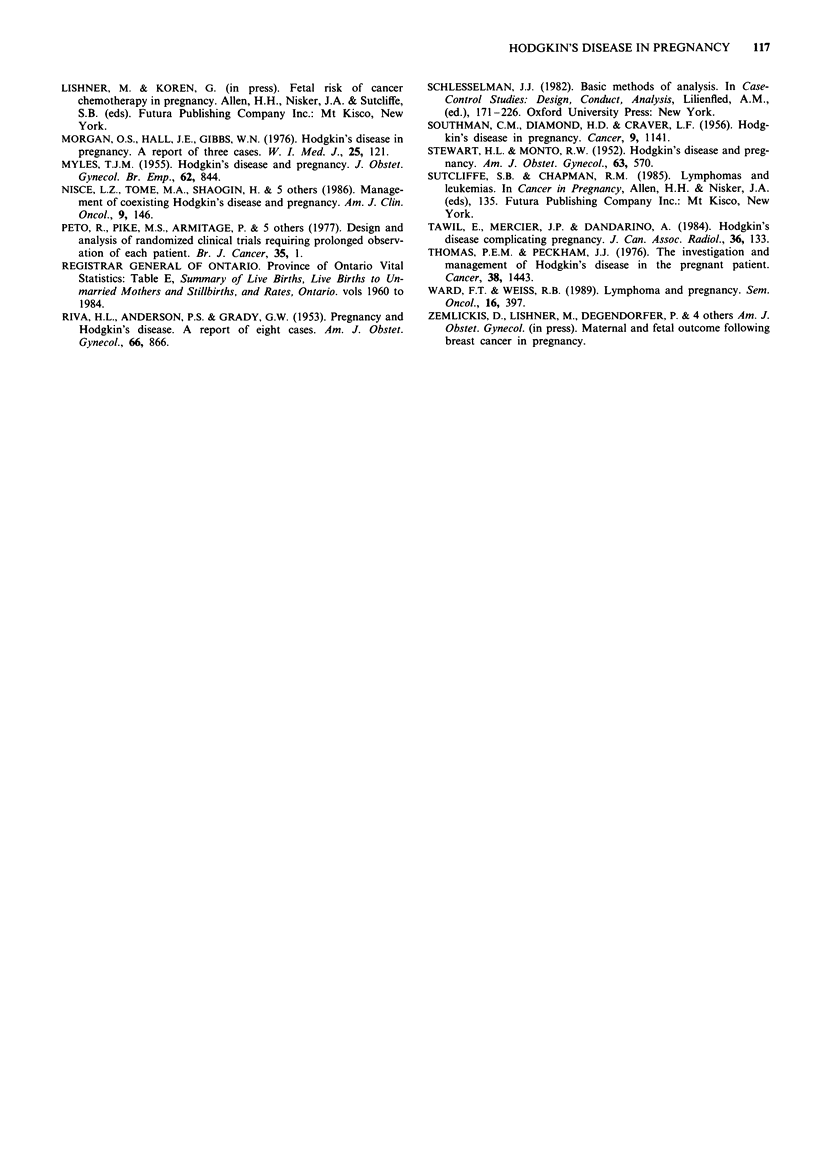# Maternal and foetal outcome following Hodgkin's disease in pregnancy.

**DOI:** 10.1038/bjc.1992.21

**Published:** 1992-01

**Authors:** M. Lishner, D. Zemlickis, P. Degendorfer, T. Panzarella, S. B. Sutcliffe, G. Koren

**Affiliations:** Department of Pediatrics, Hospital for Sick Children, Toronto, Canada.

## Abstract

The peak incidence of Hodgkin's disease occurs during the reproductive age, and its association with pregnancy is at a rate of between 1:1,000-1:6,000. We studied the effects of Hodgkin's Disease on the course and survival of 48 women who had Hodgkin's Disease and who were pregnant, and compared their outcome with non-pregnant matched women who were of similar stage of disease, age at diagnosis, and calenderic year of treatment. Twenty-year survival of pregnant women with Hodgkin's Disease was not different from that of their matched controls. Pregnant women with Hodgkin's Disease had similar distribution of stages to the controls.


Br. J. Cancer (1992), 65, 114–117 © Macmillan Press Ltd., 1992

### Maternal and foetal outcome following Hodgkin's disease in pregnancy

M. Lishner, D. Zemlickis, P. Degendorfer, T. Panzarella, S.B., Sutcliffe & G. Koren

*The Motherisk Program, Division of Clinical Pharmacology and Toxicology, Department of Pediatrics and The Research Institute, The Hospital for Sick Children and Princess Margaret Hospital, Toronto; Departments of Pediatrics, Pharmacology, Medicine, and Radiology, The University of Toronto, Canada.*

**Summary** The peak incidence of Hodgkin's disease occurs during the reproductive age, and its association with pregnancy is at a rate of between 1:1,000–1:6,000. We studied the effects of Hodgkin's Disease on the course and survival of 48 women who had Hodgkin's Disease and who were pregnant, and compared their outcome with non-pregnant matched women who were of similar stage of disease, age at diagnosis, and calendaric year of treatment. Twenty-year survival of pregnant women with Hodgkin's Disease was not different from that of their matched controls. Pregnant women with Hodgkin's Disease had similar distribution of stages to the controls.

Because the peak incidence of Hodgkin's Disease (HD) is in the age range 20–40 years, its association with pregnancy is not uncommon, occurring in 1:1,000–1:6,000 deliveries (Ward & Weiss, 1989). Early studies reported a higher frequency of relapse and lower survival rates in HD patients who were pregnant (Southman *et al.*, 1956). Later publications rejected this conclusion, claiming that pregnancy neither exacerbates the disease nor adversely affects survival (Stewart & Monto, 1952; Riva *et al.*, 1953; Barry *et al.*, 1962; Gobbi *et al.*, 1984; Tawil *et al.*, 1984; Nisce *et al.*, 1986). The view that pregnancy does not affect the course of Hodgkin's disease and the disease does not affect the course of pregnancy has become widely accepted and repeatedly stressed in reviews and textbooks (Ward & Weiss, 1989; Sutcliffe & Chapman, 1985; Becker, 1968). However, this conclusion is based on single cases or a surprisingly small number of uncontrolled studies (Barry *et al.*, 1962; Gobbi *et al.*, 1984; Tawil *et al.*, 1984; Nisce *et al.*, 1986).

It is unlikely that prospective controlled studies will ever be undertaken to further explore the interaction between HD and pregnancy; therefore, we performed an historical cohort study to evaluate the influence of pregnancy on HD and that of HD on pregnancy among women treated at the Princess Margaret Hospital (PMH) between 1958–1984. To the best of our knowledge, this is the only controlled study of HD and pregnancy. In addition, this study is unique in providing information on foetal outcome.

### Methods

All patients with histologically confirmed Hodgkin's disease, registered in the Princess Margaret Hospital between 1958–1984 were identified. PMH is an oncologic hospital serving the Province of Ontario. Cases with HD in pregnancy were identified by extracting from the database all women who had HD, where the woman was pregnant within 9 months prior to or 3 months after the first treatment. Based on the assumption that cancer would be present for some time before it could be diagnosed, 9 months was chosen to reflect the normal gestational period and 3 months was chosen arbitrarily to make the width of the time frame 1 year. The day of the first treatment was used only to extract the patients from the database because dates of diagnosis were not always recorded in the Princess Margaret Hospital (PMH) computer. For the purposes of the study, the date of diagnosis was used as a reference date after the charts had been examined. Potential cases were further screened by examining their charts to verify that pregnancy and HD occurred within the time frame stated above and to reject patients who had an ectopic pregnancy.

To study the effects of pregnancy on the course of HD, we matched women having HD in pregnancy to non-pregnant women with the disease. For each pregnant case, an attempt was made to identify three matched controls in the PMH database according to the following criteria:

(1) The control women had to be within 2 years of age of the pregnant cases at diagnosis and could not have been pregnant within 15 months prior to or 9 months after first treatment.
(2) Controls had the same Ann Arbor stage of HD as cases at diagnosis (Hermanek & Sobin, 1987).
(3) Controls had the same presence or absence of B symptoms as cases at diagnosis (fever, night sweats, and/or weight loss of more than 10% of the original weight 6 months prior to first attendance).
(4) Controls were diagnosed within 2 calenderic years of the associated case. It was assumed that controls would have similar staging procedures and treatment protocols as the cases if controls were of same age, stage, and calendaric year of diagnosis as the cases. The validity of this assumption was verified in a random sample of ten pregnant women and 23 controls and was true 96% of the time.

Data describing patient characteristics with HD including date of diagnosis, staging, and presence or absence of B symptoms were extracted from the charts of both cases and controls. Dates and types of treatment, including treatment delays, were also recorded. Obstetrical information, including date of conception, gestational age at diagnosis and at first treatment, complications in pregnancy and its outcome were also recorded.

For cases whose pregnancies continued to term or resulted in stillbirth, birth records were requested from the delivering hospital. For live births, sex and birth weight of the infant were recorded, as well as gestational age at delivery, type of delivery, foetal complications and congenital anomalies. For intrauterine death, date of diagnosis of the stillbirth and autopsy results were collected.

Live births were compared to those of infants born to women attending the Motherisk Program at The Hospital for Sick Children in Toronto, following exposure to drugs, chemicals, or radiation during the first trimester of pregnancy (Koren & MacLeod, 1986). For this analysis, each mother with HD was matched to a mother of similar age who was exposed to non-teratogenic drugs or chemicals.

### Statistical analysis

Cause-specific survival curves were produced using the Kaplan-Meier estimate (Kaplan & Meier, 1958). For the

Correspondence: G. Koren, Division of Clinical Pharmacology, The Hospital for Sick Children, 555 University Avenue, Toronto, Ontario, M5G 1X8, Canada.
This research was supported by the Motherisk Research Fund.
Received 10 May 1991; and in revised form 18 September 1991.

purposes of this paper, cause-specific survival is defined as mortality due to the disease under investigation. Any death caused by other than the disease under investigation is treated as censored. The Mantel-Haenszel logrank test was employed to test for differences between survival curves (Peto *et al.*, 1977). A chi-square test was utilised to compare the distribution of stages upon diagnosis between the cases and the non-pregnant women having HD during the reproductive age registered in the PMH database and to verify that the crtieria for matching was similar in cases and controls. The Bonferroni method was used to adjust for multiple comparisons. Fisher's exact test was used to compare the effect of stage on maternal outcome between cases and controls. Foetal outcome values between the study and control groups were compared using a two-sided Student's *t*-test for independent samples and chi-square tests whenever appropriate. The odds ratio was used to estimate the relative risk of pregnant women with HD to have a stillbirth compared to pregnant woman without cancer having a stillbirth in the province of Ontario (Schlesselman, 1982). The observed number of stillbirths was assumed to follow a Poisson distribution. Statistical analysis was performed with the aid of SAS version 5.1 and Minitab release 6.1.1.

**Figure 1** Kaplan-Meier cause-specific survival curve for Hodgkin's disease comparing women who were pregnant and had Hodgkin's disease ($n = 33$) with matched, non-pregnant controls ($n = 67$). $P = 0.6$.

### Results

Forty-eight women with HD and pregnancy were identified in the PMH database between 1958–1984. Two women had two pregnancies, each fitting in the time frame of the study, resulting in 50 pregnancies in 48 women. The mean age of women with HD was 26.1 +/− 4.9 years (median 25 years and a range of 18 to 38 years). Of the 50 pregnancies, 12 (24%) were diagnosed with HD before conception, 10 (20%) during pregnancy, and 27 (54%) were diagnosed after delivery or pregnancy termination. For one pregnancy this information was unavailable.

Treatment modalities of the 48 women included radiotherapy alone ($n = 31$), chemotherapy alone ($n = 6$), and combined radiotherapy and chemotherapy ($n = 11$). Of those women diagnosed before or during the pregnancy ($n = 22$), 16 women received radiation while pregnant, one received chemotherapy during the first trimester, and five received combination chemotherapy and radiotherapy while pregnant. One patient delayed treatment due to pregnancy. She had received radiotherapy while pregnant and delayed chemotherapy until after delivery.

Of the 48 cases, 67 matched controls were found for 33 women. Three controls per patient could not always be obtained because of the selectivity of the matching criteria. For 15 cases, matched controls could not be found. This unmatched group was not statistically significantly different from the matched group when compared by survival ($P = 0.6$).

### Maternal outcome

Using a cause-specific Kaplan-Meier survival curve, the 20-year survival of the 33 cases was compared to their 67 matched controls (Figure 1). No statistically significant difference was found between these groups ($P = 0.6$). At the time of the study, eight of the cases had died of HD while the remaining 25 were either alive or died of other causes. In the control group, 12 women died of HD.

We subsequently compared survival in the subgroup of patients who were diagnosed with HD prior to conception or during the pregnancy and had matched controls ($n = 17$) with their matched controls. No statistical difference was found ($P = 0.6$).

When the effect of individual stage of HD on maternal survival was analysed, no significant differences were found between cases and controls ($P > 0.1$ for each comparison). To evaluate whether increasing age at diagnosis had an adverse impact on maternal cause-specific survival, we compared cases above ($n = 26$) to cases below ($n = 22$) the median age of 25. No statistically significant difference was found ($P = 0.32$).

In an attempt to verify whether pregnancy affected the stage of HD upon diagnosis, we compared the distribution of stages between our cases with that of non-pregnant women younger than 38 years registered in the PHM database during the same time period (1958–1984). Of the 48 pregnant women, 12 (25%) had stage 1 disease at diagnosis, 22 (45.8%) stage 2, 8 (16.7%) stage 3, and 6 (12.5%) had stage 4. Of the 529 non-pregnant women identified in the computerised database, 79 (15%) had stage 1 disease, 257 (49%) stage 2, 106 (20%) stage 3, and 87 (16%) had stage 4. No statistically significant difference was found between these two distributions ($P > 0.25$, after adjusting for multiple comparisons).

### Pregnancy outcome

Of the 50 pregnancies studied, there were 40 deliveries (two of which were stillbirths), five miscarriages, and four therapeutic abortions. The outcome of one pregnancy was unknown.

Of the 38 live births, we were able to obtain 22 obstetrical records and two autopsy reports from the delivering hospitals (the remaining were unavailable due to unidentified delivering hospitals, destroyed birth records, or refusal to release confidential documents). However, in some additional cases, the maternal charts reported details of pregnancy outcome (Table I).

No differences were found between the babies born to women with HD when compared to the Motherisk matched controls in birth weight ($P = 0.7$), mean gestational age ($P = 0.3$) or method of delivery ($P = 0.5$). One malformation was identified: This was a child with hydrocephaly born to a mother whose HD was diagnosed before conception. She was treated only by combination chemotherapy (MOPP – Nitrogen mustard, Oncovin, Prednisone, Procarbazine) during the first trimester.

Of the 22 babies born and exposed *in utero* to HD therapy ($n = 15$), one was exposed to chemotherapy during the first trimester of pregnancy, one was exposed to chemotherapy and radiation after the first trimester, six were exposed to radiation during the first trimester of pregnancy, and seven were exposed to radiation after the first trimester.

We compared the number of stillbirths in our group (two per 40 total births) to that of the general population of Ontario (11.3 stillbirths per 1,000 total births) (Province of Ontario Vital Statistics, 1960–1984). The difference was not statistically significant ($P = 0.076$).

**Table I** Comparison of foetal outcome in mothers with Hodgkin's disease to the matched control group ($n = 38$)[a]

| | *n* | *Study babies* | *n* | *Matched contol babies* |
|---|---|---|---|---|
| Gestational age (weeks) | 29 | 39.7 +/− 1.0 | 37 | 40.0 +/− 1.8 |
| Number of preterm births (<37 weeks) | 29 | 1 | 37 | 1 |
| Birth weight (g) | 21 | 3325 +/− 529 | 37 | 3371 +/− 474 |
| Delivery method | 25 | 20 spontaneous 5 caesarean | 38 | 32 spontaneous 6 caesarean |
| Malformations | 31 | 1 case of hydrocephaly, baby died 4 h after birth | 38 | None |
| Stillbirth | 40 | 2 | 38 | None |

[a]Plus-minus values are means +/− SD.

### Discussion

The diagnosis of HD in pregnancy puts immense stress on pregnant women, their families and on physicians caring for them. Potential harm to the woman from delayed diagnosis, staging or therapy, and the risk to the baby from radiation or chemotherapy creates serious pressure and a need for prompt decisions. For such choices to be authoritative, they must be based on large experience, which is lacking in any particular centre due to the relative rareness of the combination of HD and pregnancy.

Most published studies suffer from major problems that make them difficult to interpret and cast doubt on their validity. First, some of the frequently references studies were performed decades ago (Stewart & Monto, 1952; Barry *et al.*, 1962; Kadson, 1949; Bichel, 1950; Myles, 1955). Since the diagnostic tools, staging methods, and especially treatment modalities have progressed tremendously in recent years, it is difficult to extrapolate these results to the present time. Second, most studies do not compare matched controls but rather compare pregnant women with a nonmatched cohort of nonpregnant women with HD (Barry *et al.*, 1962; Gobbi *et al.*, 1984). Such reports carry a substantial risk of bias since the groups compared may be unbalanced for significant prognostic factors and thus the analysis may be misleading. Finally, some papers provide case reports or a summary of an experience with a small number of patients (Howard *et al.*, 1978; Jacobs *et al.*, 1981; Morgan *et al.*, 1976). Although these reports are important, they cannot be used as guidelines for a rational clinical approach for these patients. In contrast to previous work, our study is the first to use a case-control method to study the outcome of pregnant women with HD. It is based on a significant number of patients that were carefully matched for the recognised and important prognostic factors of HD (Ward & Weiss, 1989). In addition, patients and controls were staged according to modern recommendations.

Our study did not detect an effect of pregnancy on survival of women with HD, as their long term prognosis was identical to that of their matched controls. Moreover, this analysis reveals that the pregnant women is not more likely to be at a higher stage (more advanced disease) than women of reproductive age in general. This indicates that pregnancy is not likely to change the biology of the tumour or to postpone diagnosis. These results are in contrast to our findings with breast cancer, where pregnant women have a significantly higher risk of being diagnosed with metastatic disease (stage 4) (Zemlickis *et al.*, in press).

Our study is the first to provide data regarding foetal outcome by analysing infants born to women with HD during pregnancy. We found that infants born to women with HD did not have a higher risk for prematurity or intrauterine growth retardation. Conversely, babies born to women with breast cancer have been shown by us to suffer from a significant small for gestational age (SGA) risk (Zemlickis *et al.*, in press). The rate of stillbirth was not statistically different from data for Ontario. This may reflect a beta error due to a small sample size, and larger numbers will be needed to confirm it.

Although various chemotherapeutic agents have been successfully administered during early pregnancy (Nisce *et al.*, 1986; Thomas & Peckham, 1976), there is compelling evidence that chemotherapy has significant likelihood of adversely affecting the baby during embryogenesis (Lishner & Koren, in press). The only infant in this series born to a patient who received chemotherapy during the first trimester (MOPP-Nitrogen Mustard, Oncovin, Prednisone, Procarbazine) had hydrocephaly and died in early infancy. In contrast, there is no evidence for teratogenic effect of chemotherapy delivered during second or third trimester of pregnancy (Koren *et al.*, 1990; Lishner & Koren, in press). In our series, one baby was exposed to chemotherapy after the first trimester and was normal at birth. Much more data are needed to define the relative foetal risk of chemotherapy during embryogenesis as well as later.

In summary, this study could not detect adverse effects of pregnancy on survival of women with HD. Similarly, pregnant women are not likely to be at a higher stage of their disease than their matched controls.

### References

BARRY, R.M., DIAMOND, H.D. & CRAVER, L.F. (1962). Influence of pregnancy on the course of Hodgkin's disease. *Am. J. Obstet. Gynecol.*, **84**, 445.

BECKER, M.H. (1968). Hodgkin's disease and pregnancy. *Radiol. Clin. N. Am.*, VI, 111.

BICHEL, J. (1950). Hodgkin's disease and pregnancy. *Acta Radiol.*, **33**, 427.

GOBBI, P.G., ATTARDO-PARRINELLO, G., DANESIMO, M. & 5 others (1984). Hodgkin's disease and pregnancy. *Hematologica*, **679**, 336.

HERMANEK, P. & SOBIN, L.H. (1987). (eds) *TNM Classification of Malignant Tumours*, 4th ed. 175–179. Springer-Verlag: New York.

HOWARD, L.C.D.R., SMITH, N. & SPAULDING, L. (19780. Hodgkin's disease and pregnancy. *South. Med. J.*, **71**, 374.

JACOBS, C., DONALDSON, S.S., ROSENBERG, S.I. & 4 others (1981). Management of pregnant women with Hodgkin's disease. *Ann. Intern. Med.*, **95**, 669.

KADSON, S.C. (1949). Pregnancy and Hodgkin's disease: with a report of three cases. *Am. J. Obstet. Gynecol.*, **57**, 282.

KAPLAN, E.L. & MEIER, P. (1958). Nonparametric estimation from incomplete observations. *J. Amer. Statist. Assoc.*, **53**, 457.

KOREN, G. & MACLEOD, S.M. (1986). Monitoring and avoiding drug and chemical teratogencity. *Can. Med. Assoc. J.*, **135**, 1079.

KOREN, G., WEINER, L., LISHNER, M., ZEMLICKIS, D. & FINEGEN, J. (1990). Cancer in pregnancy: identification of unanswered questions on maternal and fetal risks. *Obstet. Gynecol. Surv.*, **45**, 509.

LISHNER, M. & KOREN, G. (in press). Fetal risk of cancer chemotherapy in pregnancy. Allen, H.H., Nisker, J.A. & Sutcliffe, S.B. (eds). Futura Publishing Company Inc.: Mt Kisco, New York.

MORGAN, O.S., HALL, J.E., GIBBS, W.N. (1976). Hodgkin's disease in pregnancy. A report of three cases. *W. I. Med. J.*, **25**, 121.

MYLES, T.J.M. (1955). Hodgkin's disease and pregnancy. *J. Obstet. Gynecol. Br. Emp.*, **62**, 844.

NISCE, L.Z., TOME, M.A., SHAOGIN, H. & 5 others (1986). Management of coexisting Hodgkin's disease and pregnancy. *Am. J. Clin. Oncol.*, **9**, 146.

PETO, R., PIKE, M.S., ARMITAGE, P. & 5 others (1977). Design and analysis of randomized clinical trials requiring prolonged observation of each patient. *Br. J. Cancer*, **35**, 1.

REGISTRAR GENERAL OF ONTARIO. Province of Ontario Vital Statistics: Table E, *Summary of Live Births, Live Births to Unmarried Mothers and Stillbirths, and Rates, Ontario.* vols 1960 to 1984.

RIVA, H.L., ANDERSON, P.S. & GRADY, G.W. (1953). Pregnancy and Hodgkin's disease. A report of eight cases. *Am. J. Obstet. Gynecol.*, **66**, 866.

SCHLESSELMAN, J.J. (1982). Basic methods of analysis. In *Case-Control Studies: Design, Conduct, Analysis*, Lilienfled, A.M., (ed.), 171–226. Oxford University Press: New York.

SOUTHMAN, C.M., DIAMOND, H.D. & CRAVER, L.F. (1956). Hodgkin's disease in pregnancy. *Cancer*, **9**, 1141.

STEWART, H.L. & MONTO, R.W. (1952). Hodgkin's disease and pregnancy. *Am. J. Obstet. Gynecol.*, **63**, 570.

SUTCLIFFE, S.B. & CHAPMAN, R.M. (1985). Lymphomas and leukemias. In *Cancer in Pregnancy*, Allen, H.H. & Nisker, J.A. (eds), 135. Futura Publishing Company Inc.: Mt Kisco, New York.

TAWIL, E., MERCIER, J.P. & DANDARINO, A. (1984). Hodgkin's disease complicating pregnancy. *J. Can. Assoc. Radiol.*, **36**, 133.

THOMAS, P.E.M. & PECKHAM, J.J. (1976). The investigation and management of Hodgkin's disease in the pregnant patient. *Cancer*, **38**, 1443.

WARD, F.T. & WEISS, R.B. (1989). Lymphoma and pregnancy. *Sem. Oncol.*, **16**, 397.

ZEMLICKIS, D., LISHNER, M., DEGENDORFER, P. & 4 others *Am. J. Obstet. Gynecol.* (in press). Maternal and fetal outcome following breast cancer in pregnancy.